# *Acinetobacter pullorum *sp. nov., Isolated from Chicken Meat

**DOI:** 10.4014/jmb.2002.02033

**Published:** 2020-03-17

**Authors:** Arxel G. Elnar, Min-Gon Kim, Ju-Eun Lee, Rae-Hee Han, Sung-Hee Yoon, Gi-Yong Lee, Soo-Jin Yang, Geun-Bae Kim

**Affiliations:** Department of Animal Science and Technology, Chung-Ang University, Anseong 17546, Republic of Korea

**Keywords:** *Acinetobacter pullorum* sp. nov., chicken meat, taxonomy, antimicrobial resistance

## Abstract

A bacterial strain, designated B301^T^ and isolated from raw chicken meat obtained from a local market in Korea, was characterized and identified using a polyphasic taxonomic approach. Cells were gram-negative, non-motile, obligate-aerobic coccobacilli that were catalase-positive and oxidase-negative. The optimum growth conditions were 30°C, pH 7.0, and 0% NaCl in tryptic soy broth. Colonies were round, convex, smooth, and cream-colored on tryptic soy agar. Strain B301^T^ has a genome size of 3,102,684 bp, with 2,840 protein-coding genes and 102 RNA genes. The 16S rRNA gene analysis revealed that strain B301^T^ belongs to the genus *Acinetobacter* and shares highest sequence similarity (97.12%) with *A. celticus* ANC 4603^T^ and *A. sichuanensis* WCHAc060041^T^. The average nucleotide identity and digital DNA-DNA hybridization values for closely related species were below the cutoff values for species delineation (95–96% and 70%, respectively). The DNA G+C content of strain B301^T^ was 37.0%. The major respiratory quinone was Q-9, and the cellular fatty acids were primarily summed feature 3 (C_16:1_ *ω*6*c*/C_16:1_ *ω*7*c*), C_16:0_, and C_18:1_ *ω*9*c*. The major polar lipids were phosphatidylethanolamine, diphosphatidyl-glycerol, phosphatidylglycerol, and phosphatidyl-serine. The antimicrobial resistance profile of strain B301^T^ revealed the absence of antibiotic-resistance genes. Susceptibility to a wide range of antimicrobials, including imipenem, minocycline, ampicillin, and tetracycline, was also observed. The results of the phenotypic, chemotaxonomic, and phylogenetic analyses indicate that strain B301^T^ represents a novel species of the genus *Acinetobacter*, for which the name *Acinetobacter pullorum* sp. nov. is proposed. The type strain is B301^T^ (=KACC 21653^T^ = JCM 33942^T^).

## Introduction

The earliest account of *Acinetobacter* species dates back to 1911 when Beijerinck described an organism isolated from soil, originally named *Micrococcus calcoaceticus* [1]. The current genus designation was initially proposed by Brisou and Prévot in 1954, based on motility [2]. In 1968, a comprehensive survey completed by Baumann *et al*. provided sufficient data for a group species previously classified to at least 15 different genera and species and reclassified them to a single genus, for which the name *Acinetobacter* was proposed [2]. Currently, the genus *Acinetobacter,* which belongs to the class Gammaproteobacteria, is composed of 63 species with validly published names according to List of Prokaryotic names with Standing in Nomenclature, with *Acinetobacter calcoaceticus* as the type species (http://www.bacterio.net/acinetobacter.html; last accessed November 2019) and *A. baumannii* being the most clinically significant species, implicated in both nosocomial and community-derived infections [1]. This highly complex genus is widely distributed in soil, water, and animals, with members often associated with nosocomial infections—primarily aspiration pneumonia and catheter-associated bacteremia—as well as urinary tract infections [3]. The members are characteristically Gram-negative, oxidase-negative, strictly aerobic, and non-fermenting coccobacillus cells that occur in pairs under magnification [4] and exhibit twitching motility [5]. Typically, the DNA G+C content of *Acinetobacter* spp. is in the range of 34.9–47.0% [2, 6]. The major cellular fatty acids are typically C_18:1*ω*9*c*_ and C_16:0_ [7], and the predominant polar lipid is phosphatidylethanolamine [8]. The major respiratory quinone is ubiquinone Q-9 [9].

In this study, we applied a polyphasic taxonomy approach to characterize and identify an isolate from raw chicken meat and proposed it as a novel species with the name *Acinetobacter pullorum* B301^T^.

## Materials and Methods

### Bacterial Strains

Strain B301^T^ was isolated from raw chicken meat obtained from a local market (Korea). Meat samples were homogenized in 225 ml of Dijkshoorn enrichment medium [10] in a stomacher for 2 min and incubated in a shaking incubator at 30°C and 150 rpm. At 24 and 48 h of incubation, a loopful of the enrichment culture was streaked onto CHROMagar *Acinetobacter* (CHROMagar, France). The plates were incubated at 30°C for 24–48 h, monitoring the growth of typical *Acinetobacter* spp. colonies characterized by a bright salmon-red appearance.

Typical colonies of *Acinetobacter* spp. on CHROMagar *Acinetobacter* were selected for further characterization and analysis. A purified isolate was preserved in 10% skim milk solution with glycerol (3:1, v/v) at –80°C [11]. Strains from stock suspension were cultured in tryptic soy broth (TSB, BD Difco, USA) at 30°C for 12 h before downstream experimentations. The strain was deposited into the Korean Agricultural Culture Collection (KACC) and Japan Collection of Microorganisms (JCM). *A. bohemicus* CCUG 63842^T^, *A. celticus* CCUG 69239^T^, *A. gandensis* CCUG 68482^T^, and *A. calcoaceticus* KCTC 2357^T^, obtained from the Culture Collection University of Göteborg (CCUG, Sweden) and Korean Collection for Type Cultures (KCTC, Korea), were used as reference strains.

### Phylogenetic Analysis and 16S rRNA Gene Sequencing

Confirmation was done by 16S rRNA gene sequence analysis. Genomic DNA was extracted and purified using the QIAamp PowerFecal DNA kit (Qiagen, Germany) by following the manufacturer’s protocol. PCR amplification of the 16S rRNA gene was achieved using the universal bacterial primers 27F (5'-AGAGTTTGATCCTGGCTCAG-3') and 1492R (5'-GGTTACCTTGTTACGACTT-3') following initial denaturation at 95°C for 5 min; 30 cycles at 95°C for 15 sec, 58°C for 30 sec, 72°C for 40 sec, and a final extension at 72°C for 4 min [12]. Purified PCR products were sent to SolGent Co., Ltd. (Republic of Korea) for sequencing using the primers 27F (5'-AGAGTTTGA TCCTGGCTCAG-3'), 785F (5'-GGATTAGATACCCTGGTA-3'), 518R (5'-GTATTACCGCGGCTGCTGG-3'), and 1492R (5'-GGTTACCTTGTTACGACTT-3') [11]. The nearly complete 16S rRNA gene sequence was compiled and aligned with the 16S rRNA gene sequences of related type strains obtained from the EzTaxon-e server (http://www.ezbiocloud.net) using the Clustal W algorithm of MEGA-X software. Phylogenetic trees were constructed with neighbor-joining [13] and maximum-likelihood [14] algorithms using MEGA-X software. The Jukes-Cantor model was used to determine the evolutionary distance [15]. Bootstrap analysis with 1,000 replicate data sets was performed to assess support for the clusters [16].

### Analyses of Genome Sequence, Genomic DNA–DNA Relatedness, DNA G+C Content

Genomic DNA was extracted and purified using the QIAamp PowerFecal DNA kit (Qiagen, Germany) following the manufacturer’s protocol. The whole genome of strain B301^T^ was sequenced at ChunLab, Inc. (Republic of Korea) using the PacBio RS II platform. The average nucleotide identity (ANI) and digital DNA–DNA hybridization (dDDH) values were determined based on the genome sequences of strain B301^T^ and closely related species of *Acinetobacter* using the Orthologous Average Nucleotide Identity Tool (OAT software) [17] and Genome-to-Genome Distance Calculator (http://ggdc.dsmz.php/) [18], respectively. Recommended parameters and default settings were used.

Additionally, a phylogenomic tree was constructed based on 353 core genes. Protein-coding genes present in the genomes were identified using Prodigal software [19]. Sequences of the proteins in all genomes were clustered with 50% sequence identity and 80% alignment cutoffs using Linclust software [20]. The core genes were identified as the clusters that occurred as a single-copy in all strains, and were selected for phylogenetic analyses. Multiple sequence alignment was performed for each core gene using the Muscle software [21], and the resulting 353 alignments were concatenated into a single alignment. Neighbor-joining tree was reconstructed based on the distances calculated with Maximum Composite Likelihood substitution model using the MEGA-X software [22]. A maximum-likelihood tree was reconstructed with the General Time Reversible model of substitution with 5 rate categories using the IQ-Tree software [23] and was compared with the previous NJ tree. The DNA G+C content of strain B301^T^ was calculated from the whole genome shotgun project sequence.

### Phenotypic and Biochemical Tests

Phenotypic comparisons for strain B301^T^ were performed with the reference strains, *A. bohemicus* CCUG 63842^T^, *A. celticus* CCUG 69239^T^, and *A. gandensis* CCUG 68482^T^ as well as the type species of the genus, *A. calcoaceticus* KCTC 2357^T^. The Gram reaction of strain B301^T^ was examined using a standard Gram stain kit (BD Difco, USA) by following the manufacturer’s protocol. The cell morphology was examined using transmission electron microscopy (model: JEM1010) from a 48-h (30°C) culture suspension negatively stained with phosphotungstic acid. Motility was determined in motility agar [24]. Catalase activity was detected via the production of oxygen bubbles using H_2_O_2_ (3%, v/v), and the oxidase activity was detected using a commercial oxidase strip (Sigma– Aldrich, USA). The presence of flexirubin-type pigments was investigated using 20% (w/v) KOH [25]. Growth in different culture media was performed using tryptic soy agar (TSA, BD Difco), nutrient agar (BD Difco), Luria– Bertani agar (BD Difco), MacConkey agar (BD Difco), and R2A agar (MB cell, Republic of Korea) at 30°C for 7 days. The effects of temperature on the growth of strain B301^T^ were evaluated on TSA at 25, 30, and 37°C. The effects of varying pH levels on cell growth were determined using TSB, adjusted to pH 5.0–10.0 in one-unit increments using 0.1 M citrate–phosphate buffer (pH 5.0–7.0) and 0.1 M carbonate–bicarbonate buffer (pH 8.0– 10.0) before autoclaving. NaCl tolerance test for growth was performed using TSB supplemented with different concentrations of NaCl (0, 1, 2, 3, 4, and 5% w/v) at 30°C. Anaerobic growth was monitored after incubation on TSA at 30°C for 10 days using an anaerobic jar equipped with a GasPak EZ Anaerobe Container System (BD Difco). The hydrolysis of DNA was examined using DNase agar, detected by flooding with 1N HCl after incubation [26]. Starch, carboxymethyl cellulose, and cellulose hydrolysis were observed in starch agar, CMC agar, and cellulose agar, respectively, and detected by flooding with Gram iodine after incubation [27]. Hydrolysis of Tween 20 and 80 was performed using Tween agar, supplemented with 1% of either Tween 20 or Tween 80 [28]. The biochemical characteristics and enzymatic activities of strain B301^T^ and the reference strains were identified using the API ID 32 GN system (bioMérieux, France), according to the manufacturer’s instructions.

### Chemotaxonomic Analysis

The polar lipids and respiratory quinones of strain B301^T^ were extracted from freeze-dried cells harvested from 48-h colonies on TSA at 30°C [29]. The quinones were identified by HPLC (Supelcosil LC-18-S, 250 × 4.6 mm, 5 μm). The solvent used was a mixture of chloroform and methanol (2:1, v/v) with a 1.0 ml/min flow rate [30]. Polar lipids were analyzed via two-dimensional thin-layer chromatography (Merck, Germany) using two different development solvents: chloroform–methanol–water (65:25:4, v/v/v) and chloroform–acetic acid–methanol– water (80:15:12:4, v/v/v/v) [29]. The results were visualized by spraying with phosphomolybdic acid, molybdenum blue spray reagent, and ninhydrin [31].

The cellular fatty acid composition of strain B301^T^ and related type strains, including the type species of the genus, was determined. The strains were cultured on R2A agar plates at 30°C for 2 days and harvested at the exponential phase. Saponification, methylation, and extraction were performed as previously described [32]. The Sherlock Microbial Identification System (MIDI) version 6.3 and the TSBA6.21 database was used to analyze the extracts.

### Antimicrobial Susceptibility Test

The antibiotic-resistance ontologies (ARO) of strain B301^T^ and the reference strains were identified using Resistance Gene Identifier (RGI) software (http://www.truebacid.com) with the bacterial genome data. The descriptions of each ARO are as follows: OXA-133, a beta-lactamase; AAC(3)-IIb, an aminoglycoside acyltransferase; tet(39), a tetracycline efflux pump; and RlmA(II), a methyltransferase [33]. Strain B301^T^ was compared with the reference strains for susceptibility to various antimicrobial agents via the disk-diffusion assay on Mueller–Hinton agar [34] at 30°C (25°C for *A. celticus* CCUG 69239^T^) after 24-h incubation. The presence of clear zones surrounding the disk was investigated, and the diameter (mm) of the clear zone was recorded. Resistance or susceptibility to specific antimicrobial agents was determined by following the Clinical & Laboratory Standards Institute (CLSI) guidelines [35].

## Results and Discussion

### 16S rRNA Phylogeny

**Fig. 1:**
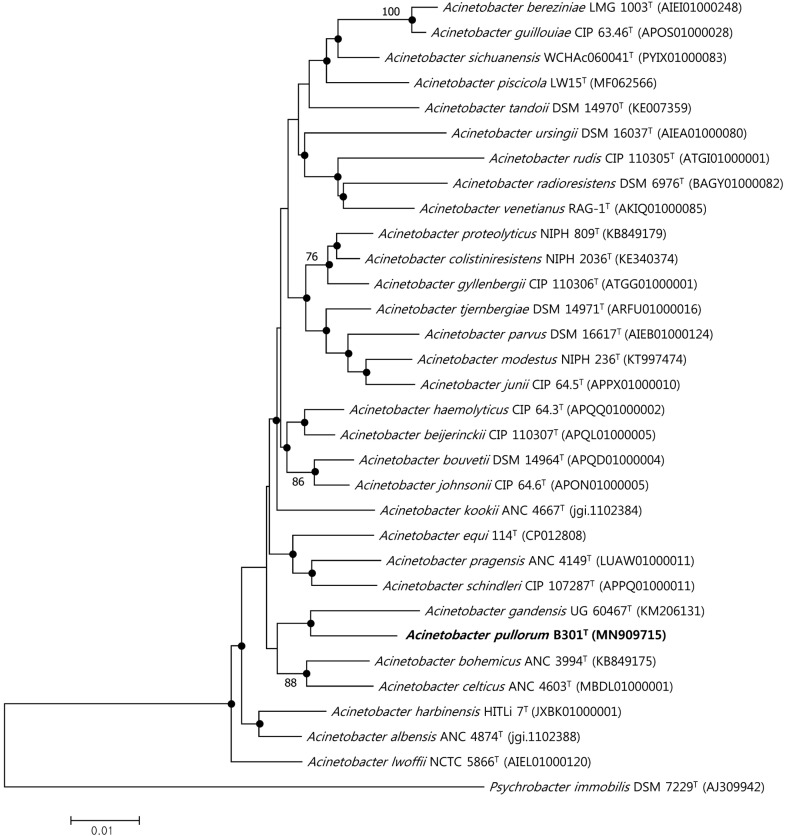
Neighbor-joining (NJ) phylogenetic tree of strain B301^T^ and related type strains based on 16S rRNA gene sequences (GenBank accession numbers are given in parenthesis). Filled circles indicate the same branches between NJ and Maximum likelihood (ML) phylogenetic tree. Numbers at nodes are bootstrap values based on 1000 resampling datasets; only values above 70% are shown. *Psychrobacter immobilis* DSM 7229^T^ was used as an outgroup. Bar, 0.01 substitutions per nucleotide position.

The length of the 16S rRNA gene sequence of strain B301^T^ was determined to be 1,462 bp. BLASTn and EzBi°loud searches revealed that strain B301^T^ had the highest sequence similarities with *A. celticus* ANC 4603^T^ and *A. sichuanensis* WCHAc060041^T^ (97.12%), and *A. piscicola* LW15^T^ (96.92%). The observed 16S rRNA sequence similarity were below the proposed species boundary of 98.7% [36]. Phylogenetic tree reconstructed by the neighbor-joining tree and compared with the maximum-likelihood algorithm (Fig. 1) revealed that strain B301^T^ forms a distinct clade with *A. gandensis* UG 60467^T^, with a neighboring clade composed of *A. bohemicus* ANC 3994^T^ and *A. celticus* ANC 4603^T^. Based on the phylogenetic tree analyses, three type strains—*A. bohemicus* CCUG 63842^T^, *A. celticus* CCUG 69239^T^, and *A. gandensis* CCUG 68482^T^—were selected for downstream biochemical and comparative analyses, including the type species of the genus, *A. calcoaceticus* KCTC 2357^T^.

### Genomic Features

The genome size of strain B301^T^ was approximately 3.103 Mb, composed of 3 contigs with 316.18× coverage (GenBank accession no. JAAARQ000000000). There were 2,840 protein-coding genes and 102 RNA genes (21 rRNA genes and 81 tRNA genes). The DNA G+C content of strain B301^T^ was 37.0% which is within the range (34.9–47.0%) reported for members of *Acinetobacter* [2, 6]. Genomic comparison of the ANI and dDDH data between strain B301^T^ and closely related species (Tables S1), *A. equi* 114^T^ (ANI: 78.7%, dDDH: 22.3%), *A. celticus* CCUG 69239^T^ (ANI: 78.2%, dDDH: 23.0%), and *A. cumulans* WCHAc060092^T^ (ANI: 77.8%, dDDH: 23.0%), revealed that the values were significantly lower than the threshold for species delineation, which is 95–96% and 70%, respectively [37]. The phylogenomic tree is presented as supplementary data (Fig. S1).

### Phenotypic and Biochemical Characteristics

Strain B301^T^ was observed as Gram-stain-negative, strictly aerobic, non-motile, oxidase-negative, and catalase-positive. Transmission electron micrographs showed a coccobacillus-shaped cell of approximately 1.5 μm in length and 0.77 μm in diameter with no appendages (Fig. S2). Growth was observed on TSA at 25–30°C and pH 6.0–9.0, with optimal growth at 30°C and pH 7.0. The isolate does not require NaCl for growth but was able to tolerate 2.0% (w/v) NaCl supplemented in TSB. Table 1 presents the phenotypic characteristics of strain B301^T^ compared with the reference strains. Moreover, Strain B301^T^ was not able to hydrolyze Tween 20, Tween 80, or gelatin, whereas the reference strains tested positive in at least one hydrolysis test. Utilization of β-alanine, citrate, glycogen, L-histidine, D-malate, L-proline, and valerate was also observed, wherein citrate and glycogen utilization are unique to strain B301^T^.

### Chemotaxonomic Characteristics

The major respiratory quinones present in strain B301^T^ were Q-9 (83.0%), Q-8 (13.0%), and Q-10 (4.0%), consistent with those of *Acinetobacter piscicola* LW15^T^ and *A. equi* 114^T^ [33]. The primary polar lipid of strain B301^T^ was phosphatidylethanolamine, along with diphosphatidyl-glycerol, phosphatidylglycerol, and phospha-tidylserine. Moreover, an unidentified aminophospholipid, was detected as a minor polar lipid (Fig. S3). The major cellular fatty acids of strain B301^T^ consist of summed feature 3 (C_16:1_ *ω*6*c*/C_16:1_ *ω*7*c*, 47.67%), C_16:0_ (22.19%), and C_18:1_ *ω*9*c* (7.95%), as presented in Table 2. Strain B301^T^ showed higher proportions of summed feature 3 and saturated fatty acids C_16:0_ and C_14:0_ than the reference strains. However, strain B301^T^ contained a significantly lower proportion of unsaturated fatty acid C_18:1_ *ω*9*c* (7.95%) compared with the reference strains (23.82–37.53%).

Results of the RGI software analysis revealed that strain B301^T^ possesses no antibiotic-resistance gene (Table S2). The absence of OXA-133, AAC(3)-IIb, tet(39), and RlmA(II) indicates susceptibility to beta-lactam antibiotics, aminoglycosides, tetracycline, and macrolide and lincosamide antibiotics. The Kirby–Bauer disk-diffusion assay showed that strain B301^T^ was highly susceptible to all antimicrobials used, with the highest zones of inhibition for imipenem (40.0 mm), minocycline (35.0 mm), and cefepime (34.0 mm), and the smallest for ciprofloxacin (26.0 mm).

The DNA G+C content, and the respiratory quinone, fatty acid, and polar lipid profiles of strain B301^T^ supported the assignment of the strain to the genus *Acinetobacter*. Distinguishing phenotypic, biochemical, and chemotaxonomic properties, highlighted in the phylogenetic placement as well as low levels of 16S rRNA gene sequence similarities and genomic indexes with other related species, suggest that strain B301^T^ represents a novel species, for which the name *Acinetobacter pullorum* B301^T^ is proposed.

### Description of *Acinetobacter pullorum* sp. nov.

*Acinetobacter pullorum* (pul.lo’rum. L. gen. pl. n. *pullorum* of chickens).

Cells of strain B301^T^ are Gram-stain-negative, strictly aerobic, and non-motile coccobacilli that are approximately 1.5 μm × 0.77 μm in size, and are oxidase-negative, and catalase-positive. Colonies are convex, smooth, cream-colored, and circular with an entire margin of approximately 1.0–2.0 mm in diameter on TSA after 2 days of incubation at 30°C. The strain grows at 0–2.0% (w/v) NaCl (optimum, 0%), in a temperature range of 25– 35°C (optimum, 30°C) and a pH range of 6.0–9.0 (optimum, pH 7.0). Respiration occurs under strict aerobic conditions. The isolate shows no hydrolysis activity. Cells assimilate β-alanine, citrate, glycogen, L-histidine, D-malate, L-proline, and valerate but not capric acid, 4-hydroxybenzoate, malonate, and propionic acid. The major respiratory quinone is Q-9. The cellular fatty acids are summed feature 3 (C16:1 *ω*6*c*/C_16:1_ *ω*7*c*, 47.67%), C_16:0_ (22.19%), and C_18:1_ *ω*9*c* (7.95%). The major polar lipids are phosphatidylethanolamine, diphosphatidyl-glycerol, phosphatidylglycerol, and phosphatidylserine. Strain B301^T^ possesses no antibiotic-resistance gene profile (OXA-133, AAC(3)-IIb, tet(39), and RlmA(II)) and is highly susceptible to a wide range of antimicrobials, including imipenem, minocycline, cefepime, ampicillin, and tetracycline. The DNA G+C content of strain B301^T^ is 37.0%. The type strain is B301^T^ (= KACC 21653^T^ = JCM 33942^T^), isolated from raw chicken meat. The GenBank Accession Numbers for the 16S rRNA gene and genome sequence are MN909715 and JAAARQ000000000, respectively.

## Supplementary material

Supplementary data for this paper are available on-line only at http://jmb.or.kr.



## Figures and Tables

**Table 1:** Differential phenotypic properties of strain B301^T^ and related species and type species of the genus *Acinetobacter*.

Characteristics	1	2	3	4	5
Temperature for growth (°C)					
Range	25-35	25-32	25-30	25-37	15-37
Optimum	30	30	25	30	30
Growth at 37°	-	-	-	+	+
Growth at 35°	+	-	-	+	+
Growth at 32°	+	+	-	+	+
pH for growth					
Range	6.0 - 9.0	7.0 - 8.0	6.0 - 8.0	6.0 - 8.0	6.0-8.0
Highest NaCl tolerance (%, w/v)	2.0	1.0	1.0	1.0	1.0
Enzyme activity					
Catalase	+	-	+	+	+
Hydrolysis:					
Tween 20	-	+	+	+	+
Tween 80	-	+	-	+	+
Liquefaction of gelatin	-	-	-	+	-
Assimilation of:					
β-Alanine	+	+	+	-	-
Capric acid	-	+	-	+	-
Citrate	+	-	-	-	-
Glycogen	+	-	-	-	+
L-Histidine	+	+	-	-	+
4-Hydroxybezonate	-	+	-	-	+
D-Malate	+	+	-	+	-
Malonate	-	+	-	-	+
L-Proline	+	+	-	+	+
Propionic acid	-	-	-	+	+
Valerate	+	+	-	+	+
DNA G+C content (%)	37	39.6	39.4	39.6	38.7^a^

Strains: 1, B301^T^; 2, *A. bohemicus* CCUG 63842^T^; 3, *A. celticus* CCUG 69239^T^; 4, *A. gandensis* CCUG 68482^T^, 5, *A. calcoaceticus* KCTC 2357^T^. +, Positive; -, Negative; All strains grow under optimum conditions of pH 7 and 0% NaCl. Data are from this study unless otherwise indicated.

^a^Data from Ho *et al.* [38]

**Table 2:** Cellular fatty acid composition of strain B301T and related species and type species of the genus *Acinetobacter*.

Fatty acids	1	2	3	4	5
C_10:0_	1.91	2.59	TR	0.84	TR
C_12:0_	4.73	2.98	5.64	7.97	5.23
C_12:0_ 2OH	1.84	TR	TR	0.54	2.09
C_12:0_ 3OH	5.56	4.31	5.10	5.70	3.92
C_14:0_	2.38	TR	0.72	0.81	TR
C_14:0_ 3OH	TR	TR	TR	TR	2.04
C_16:0_	22.19	16.12	13.05	15.04	13.10
C_16:0_ N alcohol	TR	1.59	TR	TR	0.89
C_16:1_ *ω*7*c* alcohol	TR	TR	TR	TR	1.37
C_16:1_ *ω*9*c*	TR	1.34	1.02	TR	TR
C_17:0_	TR	0.58	TR	TR	3.17
C_17:0_ iso	TR	0.51	TR	TR	0.67
C_17:1_ *ω*8*c*	0.52	TR	TR	0.75	3.69
C_18:0_	1.19	4.18	3.51	2.67	2.84
C_18:1_ *ω*9*c*	7.95	34.20	37.53	23.82	26.71
C_18:3_ *ω*6*c* (6,9,12)	TR	1.02	TR	TR	1.22
Summed feature 3*	47.67	25.88	26.40	35.57	28.97
Summed feature 8*	3.02	3.05	5.29	5.34	2.27
Total	98.96	98.35	98.26	99.05	98.18

Strains: 1, B301^T^; 2, *A. bohemicus* CCUG 63842^T^; 3, *A. celticus* CCUG 69239^T^; 4, *A. gandensis* CCUG 68482^T^; 5, *A. calcoaceticus*

KCTC 2357^T^. TR, trace (<0.5%).

*Summed features represent groups of two fatty acids that could not be separated by HPLC with the Microbial Identification System (MIDI, Inc.). Summed feature 2 consisted of C_12:0_ aldehyde; summed feature 3 consisted of C_16:1_ *ω*6*c* and/or C_16:1_ *ω*7*c*; summed feature 8 consisted of C_18:1_ *ω*7*c* and/or C_18:1_ *ω*6*c*.

## References

[1] Howard A, O'Donoghue M, Feeney A, Sleator RD (2012). Acinetobacter baumannii. Virulence.

[2] Peleg AY, Seifert H, Paterson DL (2008). *Acinetobacter baumannii*: Emergence of a successful pathogen. Clin. Microbiol. Rev..

[3] Wong D, Nielsen TB, Bonomo RA, Pantapalangkoor P, Luna B, Spellberg B (2016). Clinical and pathophysiological overview of *Acinetobacter* infections: a century of challenges. Clin. Microbiol. Rev..

[4] Rebic V, Masic N, Teskeredzic S, Aljicevic M, Abduzaimovic A, Rebic D (2018). The importance of *Acinetobacter* species in the hospital environment. Med. Arch..

[5] Bitrian M, Gonzalez RH, Paris G, Hellingwerf KJ, Nudel CB (2013). Blue-light-dependent inhibition of twitching motility in *Acinetobacter baylyi* ADP1: additive involvement of three BLUF-domain-containing proteins. Microbiology.

[6] Juni E, Brenner DJ, Krieg NR, Stanley JT (2005). Genus II. Acinetobacter Brisou and Prevot 1954. Bergey's Manual of Systematic Bacteriology.

[7] Yang C, Guo ZB, Du ZM, Yang HY, Bi YJ, Wang GQ (2012). Cellular fatty acids as chemical markers for differentiation of *Acinetobacter baumannii* and *Acinetobacter calcoaceticus*. Biomed. Environ. Sci..

[8] Luo Y, Javed MA, Deneer H, Chen X (2018). Nutrient depletion-induced production of tri-acylated glycerophospholipids in *Acinetobacter radioresistens*. Sci. Rep..

[9] Hiraishi A, Masamune K, Kitamura H (1989). Characterization of the bacterial population structure in an anaerobic-aerobic activated sludge system on the basis of respiratory quinone profiles. Appl. Environ. Microbiol..

[10] Carvalheira A, Ferreira V, Sillva J, Teixeira P (2016). Enrichment of *Acinetobacter* spp. from food samples. Food Microbiol..

[11] Han RH, Lee JE, Yoon SH, Kim GB (2020). *Acinetobacter pullicarnis* sp. nov. isolated from chicken meat. Arch. Microbiol..

[12] Baker GC, Smith JJ, Cowan DA (2003). Review and re-analysis of domain-specific 16S primers. J. Microbiol. Methods.

[13] Saitou N, Nei M (1987). The neighbor-joining method: a new method for reconstructing phylogenetic trees. Mol. Biol. Evol..

[14] Felsenstein J (1981). Evolutionary tree from DNA sequences: a maximum likelihood approach. J. Mol. Evol..

[15] Jukes TH, Cantor CR, Munro HN (1969). Evolution of protein molecules. Mammalian Protein Metabolism.

[16] Felsenstein J (1985). Confidence limits on phylogenies: an approach using bootstrap. Evolution.

[17] Lee I, Kim YO, Park SC, Chun J (2016). OrthoANI: an improved algorithm and software for calculating average nucleotide identity. Int. J. Syst. Evol. Microbiol..

[18] Meier-Kolthoff JP, Auch AF, Klenk H, Goker M (2013). Genome sequence-based species delimitation with confidence intervals and improved distance functions. BMC Bioinformatics.

[19] Hyatt D, Chen GL, LoCascio PF, Land ML, Larimer FW, Hauser LJ (2010). Prodigal: prokaryotic gene recognition and translation initiation site identification. BMC Bioinformatics..

[20] Steinegger M, Söding J (2018). Clustering huge protein sequence sets in linear time. Nat. Commun..

[21] Edgar RC (2004). MUSCLE: multiple sequence alignment with high accuracy and high throughput. Nucleic Acids Res..

[22] Kumar S, Stecher G, Li M, Knyaz C, Tamura K (2018). MEGA X: molecular evolutionary genetics analysis across computing platforms. Mol. Biol. Evol..

[23] Nguyen LT, Schmidt HA, von Haeseler A, Minh BQ (2015). IQ-TREE: a fast and effective stochastic algorithm for estimating maximum-likelihood phylogenies. Mol. Biol. Evol..

[24] Tittsler RP, Sandholzer LA (1936). The use of semi-solid agar for the detection of bacterial motility. J. Bacteriol..

[25] Fautz E, Reichenbach H (1980). A simple test for flexirubin-type pigments. FEMS Microbiol. Lett..

[26] Smith PB, Hancock GA, Rhoden DL (1969). Improved medium for detecting deoxyribonuclease-producing bacteria. Appl. Microbiol..

[27] Lal A, Cheeptham N (2012). Starch agar protocol.

[28] Plou FJ, Ferrer M, Nuero OM, Calvo MV, Alcalde M, Reyes F (1998). Analysis of Tween 80 as an esterase/lipase substrate for lipolytic activity assay. Biotechnol. Tech..

[29] Minnikin DE, O'Donell AG, Goodfellow M, Alderson G, Athalye M, Schaal A (1984). An integrated procedure for the extraction of bacterial isoprenoid quinones and polar lipids. J. Microbiol. Methods.

[30] Hiraishi A, Ueda Y, Ishihara J, Mori T (1996). Comparative lipoquinone analysis of influent sewage and activated sludge by high-performance liquid chromatography and photodiode array detection. J. Gen. Appl. Microbiol..

[31] Komagata K, Suzuki KI (1987). Lipid and call-wall analysis in bacterial systematics. Method. Microbiol..

[32] Kuykendall LD, Roy MA, O'Niell JJ, Devine TE (1988). Fatty acids, antibiotic resistance, and deoxyribonucleic acid homology groups of *Bradyrhizobium japonuicum*. Int. J. Syst. Evol. Microbiol..

[33] Alcock (2020). CARD 2020: antibiotic resistome surveillance with the comprehensive antibiotic resistance database. Nucleic Acids Res..

[34] Hudzicki J (2009). Kirby-Bauer disk diffusion susceptibility test protocol.

[35] CLSI (2019). Performance Standards for Antimicrobial Susceptibility Testing.

[36] Chun J, Oren A, Ventosa A, Christensen H, Arahal DR, da Costa MS (2016). Proposed minimal standards for the use of genome data for the taxonomy of prokaryotes. Int. J. Syst. Evol. Microbiol..

[37] Liu Y, Rao Q, Tu J, Zhang J, Huang M, Hu B (2018). *Acinetobacter piscicola* sp. nov., isolated from diseased farmed Murray cod (*Maccullochella peelii peelii*). Int. J. Syst. Evol. Microbiol..

[38] Ho MT, Weselowski B, Yuan ZC (2017). Complete genome sequence of *Acinetobacter calcoaceticus* CA16, a bacterium capable of degrading diesel and lignin. Genome Announc..

