# Supplementary material for: Acinetobacter pullorum sp. nov., Isolated from Chicken Meat
Source: J Microbiol Biotechnol. 2020 Mar 17;30(4):526–32. doi: 10.4014/jmb.2002.02033 (PMC9728200; doi:10.4014/jmb.2002.02033)
Supplement: Supplementary file 1 [file JMB-30-4-526-supple.pdf]

J. Microbiol. Biotechnol.
https://doi.org/10.4014/jmb.2002.02033

Acinetobacter pullorum sp. nov., isolated from chicken meat

Arxel G. Elnar[†], Min-Gon Kim[†], Ju-Eun Lee, Rae-Hee Han, Sung-Hee Yoon,
Gi-Yong Lee, Soo-Jin Yang, and Geun-Bae Kim*

Department of Animal Science and Technology, Chung-Ang University, Anseong 17546,
Republic of Korea

*Correspondence: Geun-Bae Kim, kimgeun@cau.ac.kr

Supplementary information

Journal of Microbiology and Biotechnology

Table S1. OrthoANI and DNA-DNA hybridization (dDDH) values between strain B301^T and type strains of genus *Acinetobacter* closely related to the strain B301^T based on 16S rRNA gene sequences derived from whole genome sequences.

Species and Strain	Accession No.	ANI Value (%)	DDH (%)
A. celticus CCUG 69239^T	MBDL01000001.1	77.89	22.2
A. sichuanensis KCTC 62575^T	PYIX00000000.2	76.81	18
A. piscicola LW15^T	NIFO00000000.1	76.63	18.9
A. johnsonii CIP 64.6^T	UFRV00000000.1	77.44	20.3
A. bohemicus CCUG 63842^T	APOH00000000.1	77.94	21.8
A. colistiniresistens NIPH 2036^T	ATGK00000000.1	74.87	16.2
A. lwoffii CIP 64.10^T	APQS00000000.1	76.48	20
A. bouvetii CIP 107468^T	APQD00000000.1	75.67	18.9
A. beijerinckii NIPH 838^T	APQL00000000.1	75.08	16.9
A. albensis ANC 4874^T	FMBK00000000.1	77.22	21.1
A. schindleri NIPH 1034^T	APPQ00000000.1	75.85	20.2
A. harbinensis HITLi 7^T	JXBK00000000.1	76.66	20.2
A. gyllenbergii NIPH 2150^T	ASQH00000000.1	74.72	15.4
A. haemolyticus CIP 64.3^T	UFRR00000000.1	75.11	16.4
A. pragensis ANC 4149^T	LUAW00000000.1	75.86	19.4
A. equi 114^T	NZ_CP012808.1	78.68	25.9
A. guillouiae CIP 63.46^T	APOS00000000.1	76.21	17.1
A. tjernbergiae CIP 107465^T	ARFU00000000.1	75.2	16.5
A. bereziniae CIP 70.12^T	APQG00000000.1	75.84	16.9
A. gandensis CCUG 68482^T	LZDS00000000.1	77.78	22.1
A. proteolyticus NIPH 809^T	APOI00000000.1	74.91	15.9
A. venetianus RAG-1^T	APPO00000000.1	75.34	16.6
A. parvus NIPH 384^T	APOM00000000.1	74.92	16.2
A. tandoii CIP 107469^T	AQFM00000000.1	76.94	19.7
A. kookii KCTC 32033^T	FMYO00000000.1	76.78	21.3
A. junii CIP 64.5^T	UFRZ00000000.1	75.27	17.1
A. radioresistens DSM 6976^T	APQF00000000.1	73.91	16.4
A. ursingii NIPH 137^T	APQA00000000.1	74.83	16.6
A. modestus NIPH 236^T	APOJ00000000.1	75.47	16.7
A. rudis DSM 24031^T	ATGI00000000.1	73.74	15.1

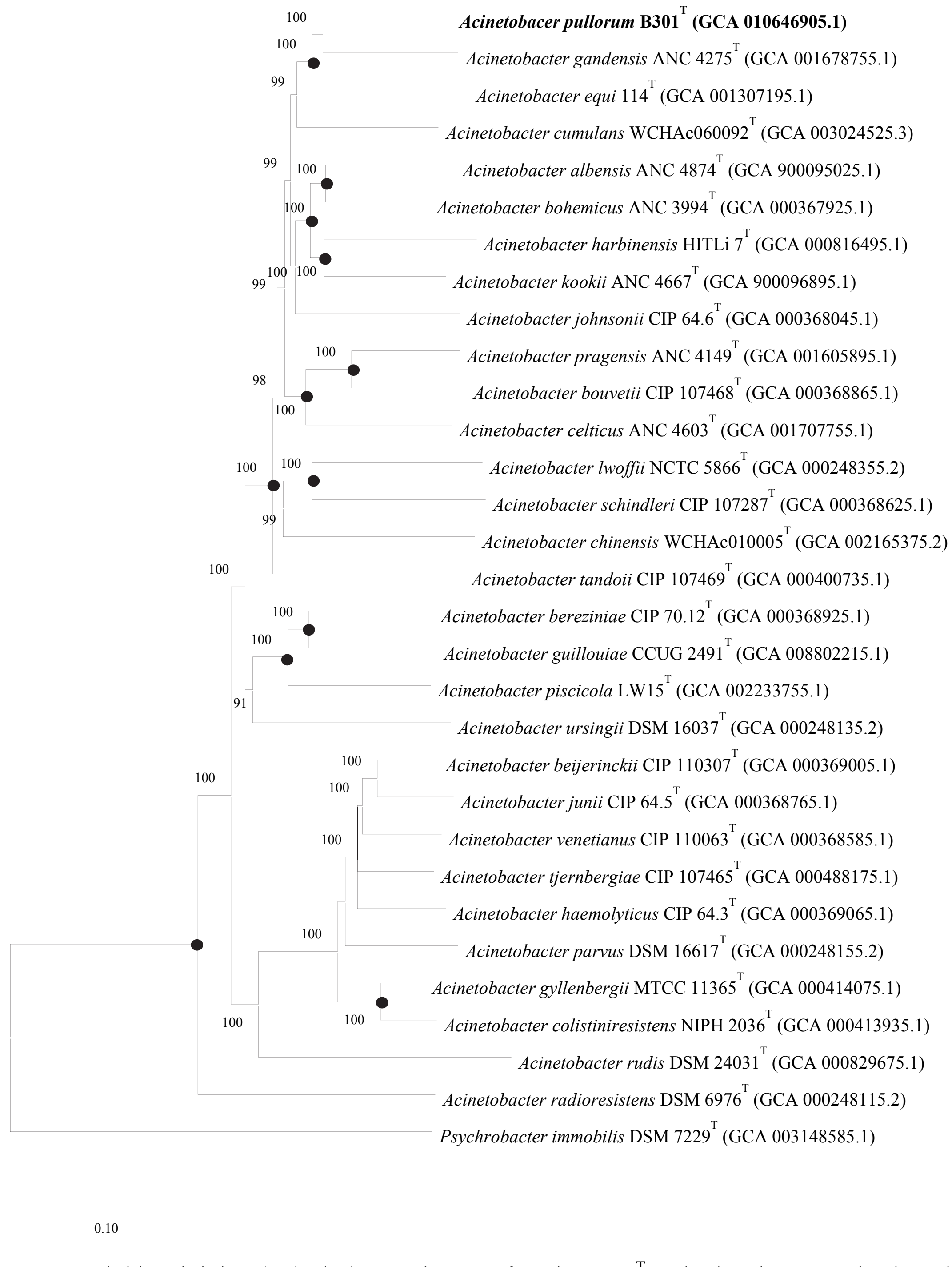

Fig. S1. Neighbor-joining (NJ) phylogenetic tree of strain B301^T and related type strains based on 353 core genes. Filled circles indicate same branches between NJ and Maximum likelihood (ML) phylogenetic tree reconstructed from the same core gene alignment. Bootstap values (expressed as percentage of 1000 replications) greater than 70% are shown. *Psychrobacter immobilis* DSM 7229^T was used as an outgroup. Bar, 0.10 substitutions per nucleotide position.

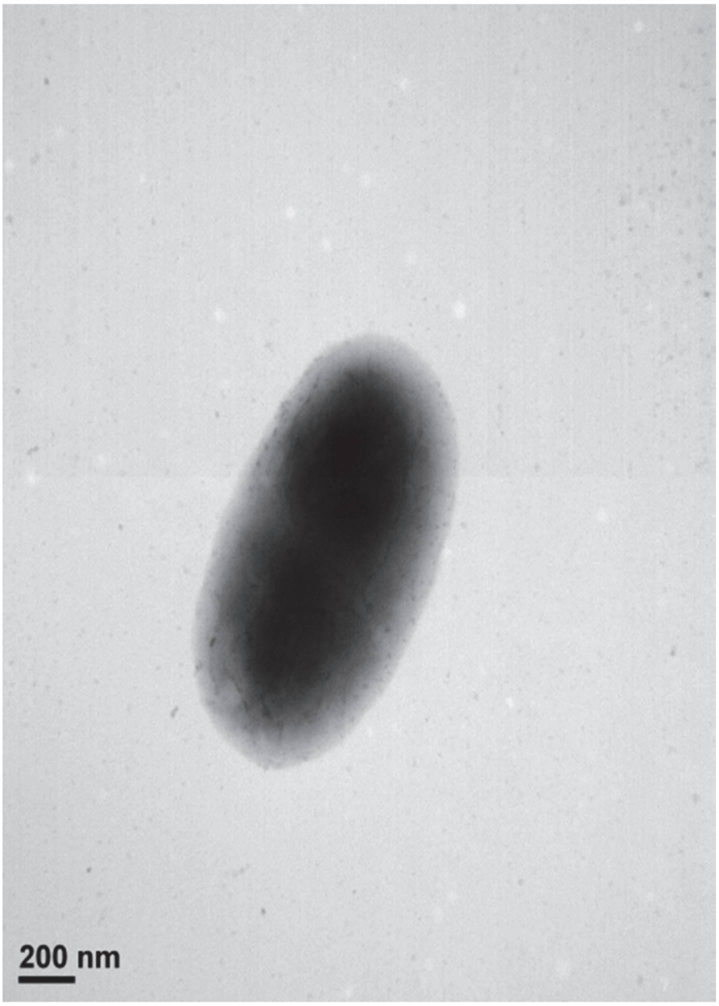

Fig S2. Transmission electron microphotograph of strain B301^{T} isolated from chicken meat. Bar, 200 nm

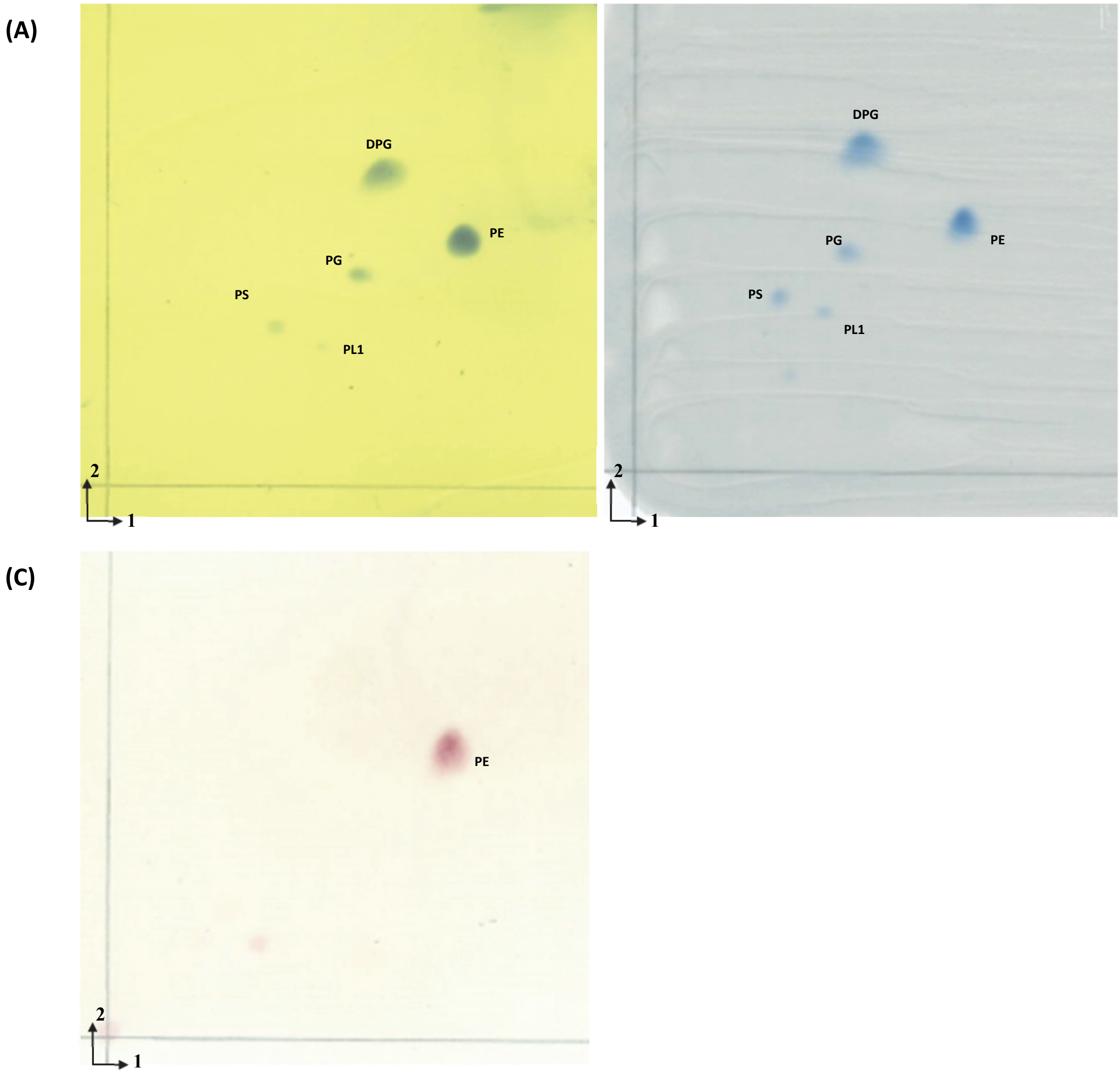

Fig S3. Two dimensional thin-layer chromatograms of the polar lipids from strains B301^T detected with reagents: phosphomolybdic acid hydrate (A), molybdenum blue spray reagent (B), ninhydrin (C). First dimension, chloroform-methanol-water (65:25:4, v/v/v); second dimension, chloroform-acetic acid-methanol-water (80:15:12:4, v/v/v/v). Abbreviations : DPG, diphosphatidylglycerol; PG, phosphatidylglycerol; PE, phosphatidylethanolamine; PS, phosphatidylserine; PL1, unidentified aminophospholipid (APL).

Table S2. Comparison of the antibiotic resistance gene profile and antibiotic sensitivity between strain B301^T and reference strains. Strains: 1, strain B301^T; 2, *A. gandensis* CCUG 68482^T; 3, *A. bohemicus* CCUG 63842^T; 4, *A. celticus* CCUG 69239^T. All data were obtained from this study. Annular radius (mm) for each antibiotic sensitivity are indicated in parentheses. +, presence; -, absence; R, resistant; I, intermediate; S, susceptible.

**Characteristic**	**1**	**2**	**3**	**4**
ARO (ID)*				
OXA-133 (3001702)	-	+	-	-
AAC(3)-IIb (3002534)	-	+	-	-
tet(39) (3000566)	-	+	-	-
RlmA(II) (3001301)	-	+	+	+
Antibiotics				
Piperacillin	S (28)	R (10)	S (25)	S (21)
Imipenem	S (40)	R (14)	S (32)	S (36)
Gentamicin	S (31)	R (12)	S (26)	S (27)
Tetracycline	S (27)	R (11)	S (28)	S (28)
Erythromyxin	S (27)	R (17)	R (18)	R (15)
Ampicillin	S (33)	S (25)	S (39)	S (31)
Ceftazidime	S (27)	S (22)	S (21)	S (21)
Cefepime	S (34)	S (20)	S (23)	S (26)
Meropenem	S (28)	I (16)	S (25)	S (28)
Amikacin	S (32)	S (28)	S (28)	S (28)
Tobramycin	S (30)	S (19)	S (26)	S (26)
Ciprofloxacin	S (26)	S (30)	S (30)	S (34)
Minocycline	S (35)	S (32)	S (31)	S (34)

*Antibiotic resistance ontology (ARO) were identified using Resistance Gene Identifier (RGI) software through TrueBacID (http://www.truebacid.com) with each genome data of strains.
